# Behavioural responses of *Konik Polski* horses to natural, familiar sound of thunderstorm, and unfamiliar similar-sounding sounds of volcanic eruption and sea storms

**DOI:** 10.1186/s12917-022-03314-4

**Published:** 2022-05-30

**Authors:** Anna Wiśniewska, Iwona Janczarek, Magdalena Ryżak, Ewelina Tkaczyk, Witold Kędzierski

**Affiliations:** 1 Department of Horse Breeding and Use, University of Life Sciences in Lublin, Akademicka 13 str, 20-950 Lublin, Poland; 2 Institute of Agrophysics, Polish Academy of Sciences, Lublin, Poland; 3 Department of Biochemistry, University of Life Sciences in Lublin, Lublin, Poland

**Keywords:** Danger, Hearing, Locomotion, Mares, Thunderclap

## Abstract

**Background:**

It is not clear, if modern *Konik Polski* horses have retained the ability to identify sounds in terms of danger. The aim of the study was to identify differences in their behaviour in response to the reproduction of volcanic eruption and sea storm sounds, assumed to be unfamiliar to these horses, as compared to their response to a thunderclap sound, considered by the horses as potentially dangerous. The study included 13 adult mares of the *Konik Polski* breed, kept under a free-range system. Their behavioural responses to the reproduction of the three natural sounds with an intensity of over 50 dB, were registered. They were analysed distance of each horse to the central point of the pasture and to the exit from the enclosure, and time and/or frequencies of elements of behaviour categorised as: increased anxiety (walking, trotting and cantering), vigilance (snoring, vocalisation, high head position, high tail position, sticking together), foraging (time of grazing), comfort (playing, examining the surroundings, sniffing), maintenance of hygiene (rubbing against objects, auto- or allogrooming, rolling) and resting. The obtained data were analysed by the Dwass, Steel and Critchlow-Fligner method using the SAS program.

**Results:**

Most of analysed elements increased in response to reproduced sounds and decreased after sounds were stop playing (*p* < 0.05), however, they were no significant differences in general response to each studied sound.

**Conclusions:**

The responses of horses to similar sounds of both known and unknown origins, i.e. the sound of a thunderstorm, sea storm and volcanic eruption, are similar. The sound stimuli applied were not too stressful for the horses.

**Supplementary Information:**

The online version contains supplementary material available at 10.1186/s12917-022-03314-4.

## Background

Since horses are inherently animals that flee predators, their senses are tuned to perceive a potential threat as quickly as possible [1]. Information about the external conditions is still perceived by the horse through its receptors, and induced stimuli are relayed via the afferent nerves to the brain where the information is proceeded. Information perceived as dangerous induces the activation of efferent nerves to mediate behavioural and physiological response of the horse. Behavioural reactions can be either active, i.e. the flight, or passive, such as freezing. The process of horse domestication has not changed their evolved behaviours of avoiding stimuli that are unfamiliar and/or recognised as dangerous [2]. Modern horses constantly analyse their surroundings, pay attention to the smallest details and often respond violently to stimuli that human senses are not able to receive [3, 4].

Depending on the breed and age, the horses’ threshold of excitability to auditory stimuli varies [5–7]. Thoroughbred horses are more sensitive to sounds than other warmblood horse breeds [8]. As claimed by researchers of horses of primitive breeds (including the *Konik Polski* horse), these animals are able to distinguish factors as being dangerous or safe with much more accuracy than horses of other breeds [9]. Wild horses developed the ability to identify a hypothetical danger to avoid wasting energy on unnecessary fleeing or even interrupting resting or foraging [10]. The factors threatening to the *Konik Polski* horse include predators, e.g. the grey wolf, humans, sudden weather changes, falling trees, etc. [11, 12]. Wild horses are known to successfully find shelter during a thunderstorm or snowstorm while avoiding other dangers [12]. Moreover, the results of studies conducted by Janczarek et al. [8, 13] indicate that horses are able to identify danger by responding to the sounds of the predators that hunted their ancestors. The *Konik Polski* horses under study did not respond to the sounds of the Arabian leopard, unfamiliar to them, or of the jackal (which poses no threat to horses), but they responded behaviourally and emotionally to the sounds of the grey wolf, whose attacks still occur today [9]. The horse’s senses are so sharp and precise that they enable, for example, the recognition of the size of a predator by the tone of the sounds it produces [14]. However, they are not scared away by a flock of birds taking flight or the sound of rustling leaves. It was also found that the sensitivity to sounds is not only a horse’s individual trait, as it is determined by the position held in the herd, e.g. stallions respond to danger more strongly and rapidly than other horses, since they have an encoded need to “warn the herd” against danger.

The sense of hearing is one of the telereceptors which enable the recognition of the surroundings from a distance, which gives the horses a huge adaptive advantage [15]. Under normal weather conditions, a horse can hear sounds from a distance of 400 m [16]. In a wind blowing at 6.7 m/s, it is able to receive sound stimuli from a distance of up to 2400 m. The mobility of the auricles makes it easier to determine the location of a sound source [17, 18].

The audible pitch range for horses is 14 Hz - 25 kHz, which exceeds the sensitivity of human hearing. Such a great audibility range allows horses to register ultrasounds, i.e. sounds with a frequency of over 20 kHz that are inaudible to humans. Moreover, low frequencies are registered by horses not only through the organ of hearing but also as vibrations of the ground, sensed through hooves or even teeth, e.g. when grazing on a meadow [19]. This enables horses to respond to incoming dangerous atmospheric phenomena (e.g. thunderstorms) or an earthquake much more quickly than humans do.

Horses have been proven to have an excellent ability to receive and remember sounds and their tones [20]. Some of these sounds are irritating or alarming to them, while others have a calming effect [5, 20]. This is because auditory stimuli are part of the cognitive units that are formed in ontogenesis, i.e. a sound registering [18]. Receiving a signal as a representation of a phenomenon in the nervous system is encoded in long-term memory, combining information from all receptors. The animal’s brain juxtaposes the information incoming on a current basis with the established cognitive unit. Stabilisation of a reaction to a signal enables the activation of an adequate behaviour after a minimum of sensory information being received. Where a stimulus falls into the range of the horse’s ontogenetic experience and is recognised as positive, then the orientation reflex is immediately inhibited [21]. In contrast, the response to a negative or alien stimulus is to flee. Therefore, both the element of novelty and the sound stimulus strength are of significance [22, 23]. An unfamiliar or very loud sound will most often trigger, during the first exposure, a strong orientation reflex that will stimulate an immediate tendency to flee [20].

The study adopted the hypothesis that modern *Konik Polski* horses have retained the ability to identify sounds in terms of danger. It was therefore assumed that the horses’ response to sounds that are similar-sounding but are familiar or unfamiliar would vary. The aim of the study was to identify differences in the behaviour of modern of *Konik Polski* horses, kept in human care in central Poland, in response to the reproduction of volcanic eruption and sea storm sounds, assumed to be unfamiliar to these horses, as compared to their response to a thunderclap sound regarded as familiar to them, and considered by the horses as potentially dangerous.

## Results

In each part of the experiment, the distance between the horses and the central point of the paddock was significantly shorter in the Before phase than in the During and After phases (Table 1). The distance between the horses and the paddock exit was significantly greater before the sound signal reproduction (Before) than during the sound signal reproduction (During) and immediately after the sound signal reproduction (After). No significant differences depending on the sound type were noted.

**Table 1:** Average distances (m) between the horses and the selected points of the paddock during three the study (means ± SD)

Phase	Volcano (Volcanic eruption sound)	Thunder (Thunderclap sound)	Sea storm (The sound of roaring waves and the wind during a sea storm)
The distance between the horses and the central point of the paddock			
Before	12.81 ± 7.72 a	12.82 ± 7.69 a	12.94 ± 7.81 a
During	23.92 ± 4.11 b	23.92 ± 4.11 b	24.44 ± 3.53 b
After	27.77 ± 7.08 b	26.72 ± 4.12 b	27.61 ± 4.18 b
The distance between the horses and the exit from the paddock			
Before	39.92 ± 5.53 a	39.83 ± 5.58 a	37.54 ± 6.92 a
During	6.94 ± 3.97 b	7.91 ± 3.92 b	7.55 ± 2.34 b
After	2.45 ± 0.44 c	3.45 ± 0.23 c	3.12 ± 0.66 c

Phases: Before - before the sound signal reproduction, During - during the sound signal reproduction, After - after the sound signal reproduction. The mean values denoted by different letters (a, b, c: between the phases) differ significantly at *P* ≤ 0.05. No significant differences depending on the sound type were noted

For all the test sounds, the duration of walking was significantly longer in Before phase than in the During and After phases (Table 2). Moreover, in Before phase, the duration of walking was longer before the reproduction of sea storm sounds than before the reproduction of the remaining sounds. In the After phase, the duration of walking was the longest after the reproduction of thunderclap sounds, and the shortest after the reproduction of sea storm sounds. The duration of trotting increased significantly in the During phase of each part of the experiment and then decreased in the After phase. However, only after the reproduction of the volcanic sounds, this value was significantly higher than the value in the Before phase. In all phases, significant differences occurred depending on the sound type, e.g. for the sea storm in the During phase, the duration of trotting was significantly longer than for the volcano, and in the After phase, a significantly higher value was observed after the reproduction of thunderclap sound. The duration of cantering, just like the duration of trotting, was significantly longer in the During phase of the experiment than in the Before and After phases. Moreover, the duration of cantering in the During phase was longer for the volcanic sound than in response to the thunderclap sound. In the After phase, the duration of cantering in response to volcano and thunderstorm sounds was significantly shorter than that recorded in response to the sea storm sounds and was shorter than in the Before phase.

**Table 2:** Time of walking, trotting and galloping (s) in response to the tested signals (means ± SD)

Phase	Volcano (Volcanic eruption sound)	Thunder (Thunderclap sound)	Sea storm (The sound of roaring waves and the wind during a sea storm)
Walk			
Before	37.00 ± 14.18 ax	30.71 ± 17.28 ax	56.00 ± 17.20 ay
During	3.20 ± 13.79 bx	5.61 ± 2.84 bx	2.81 ± 3.88 bx
After	7.12 ± 4.84 bx	11.85 ± 3.61 cy	2.45 ± 2.27 bz
Trot			
Before	11.02 ± 5.16 ax	32.31 ± 16.87 ay	48.70 ± 12.07 az
During	62.64 ± 11.07 bx	72.20 ± 11.48 bxy	81.22 ± 11.98 by
After	31.91 ± 11,52 cx	45.00 ± 13.58 ay	37.71 ± 14.95 axy
Canter			
Before	8.52 ± 5.47 ax	6.62 ± 2.84 ax	9.90 ± 6.46 ax
During	19.23 ± 8.07 bx	10.82 ± 4.23 by	13.81 ± 6.32 bxy
After	4.73 ± 1.82 cx	2.63 ± 1.81 cx	9.12 ± 5.94 ay

Phases: Before - before the sound signal reproduction, During - during the sound signal reproduction, After - after the sound signal reproduction. The mean values denoted by different letters (a, b, c: between the phases; x, y, z: between the test sounds) differ significantly at *P* ≤ 0.05

Of the five traits from the category “vigilance”, the frequency of snoring remained unchanged in successive phases of the experiment, while statistically significant differences occurred depending on the type of the test sound (Table 3). The frequency of “vocalisation” was significantly higher than the others only in the After phase in response to sea storm and thunder sounds. Significant differences between the phases as regards the “high tail position” were only noted in response to the thunderclap sound, while during and after the sound emission, this behaviour was not observed. The values of this trait were higher than the other ones mainly during the reproduction of sea storm sounds. The frequency of the occurrence of high head position decreased in the During phase in response to the volcano and sea storm sounds. In the After phase, this trait value increased, in relation to the previous phase, after the reproduction of the volcano sound and remained unchanged for the thunderclap and sea storm sounds. As regards the trait “sticking together”, no significant differences were noted in response to volcano sound. On the other hand, in response to the thunderclap sound and sea storm sound, the time the horses “sticking together” in the During and After phases 3 were significantly lower than those in the Before phase. For the sea storm, the values of this trait were significantly higher than the remaining traits noted in all three phases.

**Table 3:** Time of elements of behaviour categorised as vigilance (s) in response to the test signals (means ± SD)

Phase	Volcano (Volcanic eruption sound)	Thunder (Thunderclap sound)	Sea storm (The sound of roaring waves and the wind during a sea storm)
Snoring			
Before	0.00 ± 0.00 ax	0.76 ± 0.72 ay	2.07 ± 1.38 az
During	0.00 ± 0.00 ax	0.31 ± 0.48 ax	2.07 ± 0.86 ay
After	0.00 ± 0.00 ax	0.46 ± 0.51 ay	3.38 ± 1.26 az
Vocalisation			
Before	0.46 ± 0.51 ax	0.00 ± 0.00 ay	0.15 ± 0.37 axy
During	0.76 ± 0.43 ax	0.00 ± 0.00 ay	0.23 ± 0.43 axy
After	0.76 ± 0.59 ax	0.69 ± 0.48 bx	0.92 ± 0.64 bx
High head position			
Before	0.23 ± 0.43 ax	0.61 ± 0.51 ay	1.00 ± 0.00 ay
During	0.00 ± 0.00 ax	0.00 ± 0.00 bx	1.46 ± 1.05 ay
After	0.00 ± 0.00 ax	0.00 ± 0.00 bx	1.00 ± 0.58 ay
High tail position			
Before	1.23 ± 0.72 ax	1.85 ± 1.07 ax	4.92 ± 1.32 ay
During	0.54 ± 0.66 bx	1.23 ± 0.60 az	3.38 ± 1.42 by
After	1.46 ± 0.78 ax	1.62 ± 0.87 ax	3.15 ± 1.82 by
Sticking together			
Before	0.00 ± 0.00 ax	1.31 ± 0.75 ay	2.54 ± 1.13 az
During	0.00 ± 0.00 ax	0.54 ± 0.52 by	1.69 ± 0.95 bz
After	0.31 ± 0.48 ax	0.85 ± 0.69 bx	1.85 ± 0.90 by

Phases: Before - before the sound signal reproduction, During - during the sound signal reproduction, After - after the sound signal reproduction. The mean values denoted by different letters (a, b: between the phases; x, y, z: between the test sounds) differ significantly at *P* ≤ 0.05

The total duration of expressing the behaviour categorised as “vigilance” in relation to the test sounds was significantly longer in the During and After phases than in the Before phase (Table 4). When examining the response to the volcano, this value in the Before phase was significantly lower than the values noted when examining the responses to the thunderclap and sea storm sound reproduction. In the After phase, the value of this parameter was significantly lower after the emission of the thunderclap sound than for the volcano and sea storm sounds.

**Table 4:** Total duration (s) of behaviour categorised as “vigilance” in response to the test sounds (means ± SD)

Phase	Volcano (Volcanic eruption sound)	Thunder (Thunderclap sound)	Sea storm (The sound of roaring waves and the wind during a sea storm)
Before	12.11 ± 7.53 ax	55.12 ± 13.33 ay	50.70 ± 21.7 ay
During	124.60 ± 10.20 bx	115.50 ± 19.50 bx	123.10 ± 24.9 bx
After	152.12 ± 33.24 bx	117.95 ± 14.91 by	148.91 ± 23.2 bx

Phases: Before - before the sound signal reproduction, During - during the sound signal reproduction, After - after the sound signal reproduction. The mean values denoted by different letters (a, b: between the phases; x, y, z: between the test sounds) differ significantly at *P* ≤ 0.05

In the category of behaviours aimed at maintaining body condition, the frequency of rubbing against objects only changed in response to the thunderclap sound in the After phase (Table 5). Moreover, significant differences were noted in response to individual sounds in all phases: in the Before phase, the trait did not occur for sea storms, and it took the highest value when examining the effects of the volcano sound. In the During and After phases of testing the response to sea storms, this trait was again absent. With regard to the volcano and thunderclaps, the frequency of its expression remained at a similar level. The frequency of grooming did not differ significantly between the phases, and was often not recorded at all. The frequency of rolling decreased in the During phase compared to the Before phase, and remained at a very low level after the emission of volcanic eruption sound, and increased to the values noted in the Before phase when examining the effects of thunderclaps and sea storms. It was also noted that the values of this trait during each phase of the volcanic sound impact were significantly higher than those noted at the time of testing the response to a sea storm or thunderclaps.

**Table 5:** The frequency of behaviour categorised as maintenance of hygiene in response to the test sounds (means ± SD)

Phase	Volcano (Volcanic eruption sound)	Thunder (Thunderclap sound)	Sea storm (The sound of roaring waves and the wind during a sea storm)
Rubbing against objects and Scratching against an object			
Before	1.31 ± 0.63 ax	0.54 ± 0.52 ay	0.00 ± 0.00 az
During	1.15 ± 0.90 ax	0.38 ± 0.65 ax	0.00 ± 0.00 ay
After	1.23 ± 0.75 ax	2.00 ± 1.22 bx	0.00 ± 0.00 ay
Autogrooming or allogrooming			
Before	0.54 ± 0.52 ax	0.00 ± 0.00 ay	0.00 ± 0.00 ay
During	0.23 ± 0.43 ax	0.00 ± 0.00 ay	0.00 ± 0.00 ay
After	0.31 ± 0.48 ax	0.31 ± 0.48 ax	0.00 ± 0.00 ay
Rolling on the ground			
Before	2.54 ± 0.97 ax	0.54 ± 0.52 ay	0.53 ± 0.52 ay
During	1.46 ± 0.78 bx	0.00 ± 0.00 by	0.00 ± 0.00 by
After	0.92 ± 0.64 bx	0.46 ± 0.51 ay	0.54 ± 0.52 ay

Phases: Before - before the sound signal reproduction, During - during the sound signal reproduction, After - after the sound signal reproduction. The mean values denoted by different letters (a, b: between the phases; x, y, z: between the test sounds) differ significantly at *P* ≤ 0.05

As regards the other analysed behaviours, the duration of feed intake in the Before phase was significantly longer than the time noted for the two remaining phases (Table 6). Moreover, in the Before phase, this duration was significantly longer at the time of testing the volcanic sound impact than before the emission of the thunderclap and sea storm sounds. The duration of comfort behaviour manifestation decreased in the During and After phases compared to the Before phase, particularly after the emission of thunderstorm and sea storm sounds. Significant differences occurred when examining the responses to individual sounds: the duration of comfort behaviour in relation to the volcano in the Before and During phases was significantly longer than that during the emission of the remaining sounds. In the After phase, these behaviours were not noted in response to the emission of sea storm sounds. The duration of resting in the During phase increased transiently in response to the thunderclap sound, increased and remained at a higher level in the After phase in response to the sea storm sound while it remained unchanged in response to the volcano sound. Moreover, in the Before phase, the highest value was noted on the day of volcanic sound emission, while the lowest value was registered on the day of sea storm sound reproduction. elements of behaviour categorised as “comfort”.

**Table 6:** The frequency of foraging, comfort behaviour and resting in response to the test sounds (means ± SD)

Phase	Volcano (Volcanic eruption sound)	Thunder (Thunderclap sound)	Sea storm (The sound of roaring waves and the wind during a sea storm)
Foraging (grazing)			
Before	179.75 ± 52.1 ax	132.65 ± 53.19 ay	125.82 ± 30.02 ay
During	50.45 ± 39.22 bx	55.55 ± 47.11 bx	37.45 ± 22.23 bx
After	66.31 ± 26.09 bx	71.51 ± 63.32 bx	48.95 ± 33.92 bx
Comfort: playing, examining the surroundings, sniffing			
Before	23.00 ± 9.48 ax	4.00 ± 3.53 ay	1.10 ± 1.79 ay
During	15.00 ± 12.88 ax	2.30 ± 2.50 by	0.00 ± 0.00 by
After	2.32 ± 2.41 bx	2.21 ± 0.71 bx	0.00 ± 0.00 bx
Resting			
Before	29.01 ± 14.30 ax	15.00 ± 5.27 ay	9.00 ± 9.66 az
During	22.50 ± 11.37 ax	26.51 ± 11.07 bx	41.20 ± 25.07 by
After	26.03 ± 7.75 ax	16.51 ± 8.83 ay	32.00 ± 15.42 bx

Phases: Before - before the sound signal reproduction, During - during the sound signal reproduction, After - after the sound signal reproduction. The mean values denoted by different letters (a, b: between the phases; x, y, z: between the test sounds) differ significantly at *P* ≤ 0.05

## Discussion

The study analysed whether the horses responded the same to familiar or unfamiliar sounds of a similar tone and maximum intensity noted at 1 kHz, which, according to Timney and Macuda [17], is the lower limit of the maximum hearing sensitivity in horses. The horses’ responses to the sound of a volcanic eruption, a sea storm and a thunderclap were preceded by the analysis of selected behavioural traits during their undisturbed stay in the paddock (the Before phase), which was regarded as the control part of the experiment. The results from three tests of the experiment proved to be comparable only in terms of the horses staying in the same paddock area. The remaining behavioural traits analysed in the Before phase were significantly different, even though the environmental conditions prevailing during the experiment were comparable. These differences concerned the duration of walking and trotting and all the traits from the following categories: vigilance, maintenance of body condition, foraging, comfort and resting. It can be concluded that the horses’ behaviour within the enclosure is not schematic, which is most probably influenced by various interactions between individuals. However, it was not observed that the horses expected the sound signal that they had heard on the previous day of the experiment. Varied behaviour of the horses during their stay in the paddock was also noted by Janczarek et al. [24]. As Morris [25] points out, one of the reasons for this variation is the mood of the animals which may change on a daily basis.

The results of the behavioural response testing changed immediately after the application of sound signals. Analysis of the distance between the horses and the point in the paddock being most distant from the sound sources, and of the distance between the horses and the exit from the paddock, changed under the influence of the reproduction of all sound signals, and this situation persisted even after the end of sound reproduction. However, based on this parameter it was not possible to identify the sounds that the horses responded to more or less intensely. For each test sound, the horses moved away from the sound sources while approaching the exit situated in front of the stable. Moving away from a sound that is probably irritating to the horses’ hearing can be explained by the great range of the frequency of sounds audible to them, given the considerable sensitivity of the horses’ hearing [26]. However, heading towards the exit but remaining some distance from the gate is difficult to explain. Perhaps it was preparation to flee from irritating sounds, or the result of a situation recognised as a life-threatening possibility, or both at the same time. As indicated by a study conducted by Erber et al. [27], the introduction of modifications in a place safe for horses generates stress symptoms.

The location in the paddock, determined during the sound reproduction, was maintained even after the end of sound reproduction, which may indicate that horses remain vigilant even after the stimulus regarded as dangerous has ceased. Leiner and Fendt [28] argue similarly based on the measurements of the time of habituation in relation to a new object. This fact can also be the first indication that the horses’ sense of hearing generalises sounds that sound similar. A similar view was taken by Timney and Macuda [17], who suggested that horses are unable to use binaural intensity difference cues.

Moreover, the results showed that during the reproduction of sounds, the duration of trotting and cantering was longer, while the duration of walking decreased, which may also indicate that the stimuli used in the experiment were not identified by the horses as positive [29, 30]. The increase in locomotor activity should therefore be regarded as a clear response to the stimulus. However, just like when analysing the distance between the horses and the sound source and the exit gate, in this case, it was also not possible to determine the variation in the horses’ responses to individual sounds. While it is true that during the reproduction of sea storm sounds the duration of trotting was longer than that during the reproduction of volcano sounds, in the case concerned, the situation could have been due to the horses’ increased locomotor activity on that day of the experiment, and even before the stimulus was applied, which is difficult to explain. However, the duration of cantering was found to be longer in response to the volcanic eruption sounds than to the thunderclap sound. Moreover, after the end of the stimulus emission, the time spent cantering was shorter in response to the thunderclap sound than to the remaining sounds. One might be tempted to conclude that the sound of a thunderstorm, familiar to horses, triggered lower emotional arousal expressed by the shift from walking to cantering than that caused by unknown sounds of volcanic eruptions and sea storms.

Analysis of the results of individual behavioural trait measurements proved difficult to interpret. In general, different sound signals caused different changes in the frequency of the occurrence and duration of the behaviours under analysis. In the group of traits categorised as “vigilance”, snoring occurred most frequently during the reproduction of sea storm sounds; vocalisation increased after sea storms and thunderclaps without being affected by the volcano sounds; high tail position clearly lowered in response to thunderclap sounds; high head position lowered only during the emission of volcano sounds; and the frequency of sticking together decreased only during the emission of thunder sounds. The analysis of the presented results is hampered by the large variation in the values of analysed traits before the start of the emission of a particular sound. In general, the traits being discussed, i.e. the high head position or sticking together intensify in the face of danger [31, 32]. It is likely that the individual differences in presenting individual behaviours by the horses in response to the sound type being tested resulted in a lack of uniformity of the results obtained.

However, an analysis of the total time of expressing vigilance showed a significant increase in this parameter value during and after the reproduction of individual sounds. However, no differences in the responses to individual sounds were demonstrated. The hypothesis put forward in this paper about the horses’ precise response to sound stimuli (which allows them to recognise and select a familiar sound and, at the same time, to qualify an unfamiliar sound as an actual threat) is, at this stage of the discussion, difficult to confirm. However, the previously mentioned horses’ responses to new aural stimuli, i.e. the sounds of sea storm and volcanic eruption (which triggered a specific response similar to the response to the thunderclap sound, which was familiar to horses and potentially dangerous), also cannot be excluded. The reason for obtaining non-diversified results may also be completely different. The horses responded similarly to all three sounds, as in the frequency range < 1 kHz, the sound intensity level was almost the same at all tested frequencies. This is in line with the literature data, according to which low frequencies are well perceived by horses not only through the ears but also through the hooves and even the teeth, e.g. when grazing in a meadow [19]. The sounds differed slightly in intensity at individual frequencies >1 kHz, but the shape of the spectrum was the same. The sea storm sounds had intensity levels approx. 10 dB higher than the thunderstorm sounds. A similar shift towards higher intensity levels was observed for the volcano compared to the storm sounds; however, no major significance was observed for the horses’ behaviour. These results are partially consistent with those published by Marshall [33], who noted that since with the increasing sound stimulus intensity, the delay in responding to a stimulus was shortened, therefore the speed of the horses’ response to sound stimuli increased with an increase in the sound loudness. At this stage of the research, unambiguous interpretation of the horses’ response to the test sounds appears to be impossible.

The obtained results were also ambiguous for the manifestation of behaviour qualified as the maintenance of body condition. Of all the analysed behaviours, only the frequency of rolling decreased in response to the sounds being reproduced. For the volcano sound, the response also persisted after the end of stimulus emission. However, it is worth noting that on the day of the volcano sound reproduction, in the phase before sound reproduction, the horses rubbed against objects, groomed or rolled much more often than on other test days. The actual reproduction of the volcanic eruption sounds or the two other sounds simply did not change this behaviour type significantly. In general, elements of this behaviour occurred when animals are under optimal environmental conditions, in locations well known to them and regarded as safe [34, 35]. The frequency of such behaviours decreases in the face of danger [36].

However, the horses’ response to the reproduction of sounds is indicated by the duration of feed intake, which decreased significantly both during and after the sound reproduction. Horses graze when they do not feel threatened and are under optimal environmental conditions [37]. No differences related to the signal type were noted, although before the reproduction of volcanic eruption sounds, the duration of grazing was significantly longer than that noted during the reproduction of the two remaining signals. Therefore, at that time this duration decreased the most, which can indirectly indicate that the response to the volcanic eruption sounds was the strongest. This suggestion, however, is not confirmed by the duration of resting or comfort behaviour, especially as significant differences between these parameters occurred before the reproduction of sounds. Nevertheless, since the duration of resting did not change (and even increased), it theoretically indicates increasing relaxation in the horses rather than their anxiety. It is therefore not known whether the horses were not able to differentiate the sounds being reproduced or whether other factors, e.g. low responsiveness of the test horse breed, were decisive. Primitive horse breeds are known to be much less responsive than hot-blooded horses [38]. According to Janczarek et al. [8], there are differences in emotional excitability even between warmblood horses. It is known that a higher proportion of thoroughbred horses in a pedigree produces higher responsiveness to new stimuli, including sound stimuli.

It should also be noted that the described experiment used only sound signals, whereas in nature these signals accompany other environmental changes registered by all the senses, not only by hearing, e.g. increased wind intensity and cloudiness, changes in atmospheric pressure, rain, lightning, seismic activity, the anxiety of other animals, and probably many more. Perhaps the reproduction of a particular sound, with no visual effects or other phenomena accompanying a thunderstorm or an earthquake, was a stimulus insufficient to classify the sound as a sign of impending danger.

To sum up, the responses of horses to similar sounds of both known and unknown origins, i.e. the sound of a thunderstorm, sea storm and volcanic eruption, with an intensity of over 50 dB, are similar. These aural stimuli make the horses move away from the sound sources and approach the pasture exit. Locomotor behaviour changes as well; more specifically, the duration of trotting and cantering increases while the duration of walking decreases. However, behaviour categorised as vigilance, comfort, resting or grazing did not change significantly, which may indicate that the sound stimuli applied are not too stressful for the horses. Interpretation of the results is also hindered by partially differentiated grazing behaviour that is not disturbed by sounds.

Limitations: in general, this is the first study focused on horse behavioural response to natural sounds, thus, there is lack of similar studies, hence it was not possible to compare our results with others. The reactions of horses to sounds emitted from a recorder were studied, it was not previously investigated whether for horses they are sounds identical to natural ones. The study was performed in field conditions, thus, some uncontrolled factors (like olfactory signals and sounds inaudible to humans) could influence the horse behaviour. Moreover, the studied herd of horses included only pregnant mares. It is possible that the presence of a leading stallion in the herd may affect the mare’s reactivity. The pregnancy could also influence the mare’s behaviour, however, to the best of the authors’ knowledge, there are no scientific reports documenting differences in the mares response to sound stimuli depending on being or not being pregnant. Therefore, the obtained results should be considered with caution, and further research is needed to compete explanation of how horses react to natural sounds.

## Conclusion

The responses of the *Konik Polski* horses, kept under a free-range system, to similar sounds of both known and unknown origins, i.e. the sound of a thunderstorm, sea storm and volcanic eruption, are not differentiated. Generally, the sound stimuli applied are not too stressful for the horses. Due to the inconclusive results obtained, the research should be continued using other sounds, including those generated by human activities.

## Methods

### Horses

The study included 13 breeding mares of the *Konik Polski* breed, kept under a free-range system with permanent access to a stable and a meadow-and-forest paddock with an area of 10 ha (51°18′37.1″N, 22°58′25.2″E). The forestation rate was approx. 10% of the area. In the paddock, there was also a natural water reservoir for the horses to drink from. The stable building (of the run-pen type) was open at all times. The stable floor was bedded with wheat straw on a daily basis. Hay baskets were mounted on the long walls, with hay being offered *ad libitum*.

Each mare had been staying in the described breeding station for at least 36 months before the start of the experiment. A stallion was periodically allowed into the herd for reproductive purposes only. An annual veterinary check of the horses’ health was also carried out, and hoof horn correction was performed four times a year. Individuals with symptoms of an illness or injury were placed in the stable for the duration of treatment. The mares’ age at the time of the study was 5–10 years. During the study, they had no offspring with them and were between the fourth and sixth month of gestation. During the study, no symptoms of disease were observed in the herd.

### The course of the experiment

The experiment was conducted in a fenced-off part of the pasture with an area of 60 x 60 m. The entrance to the fenced-off area was situated 100–120 m from the entrance to the stable. The horses were let into the enclosure 60 minutes before the start of the actual experiment involving the recording of the horses’ behaviour in response to the reproduction of recorded selected natural sounds. First, the volcanic eruption (volcano) sound was reproduced as a signal unfamiliar to the horses under study due to their geographical position. After a 10-day break in the experiment, the study examined responses to the sound of a thunderclap (thunderstorm): a signal was familiar to horses, as for the last 12 months before the start of the experiment, 17 storms with atmospheric discharges, downpour, or hail and strong winds with a velocity of up to 24 m/s had occurred over the horses’ place of stay [39]. During thunderstorms, the horses were never locked in the stable. After another 10-day break, horses’ responses to the sounds of sea storm with a wind force equal to 10 on the Beaufort scale (sea storm: a signal unfamiliar to the horses due to their geographical position).

**Fig. 1:**
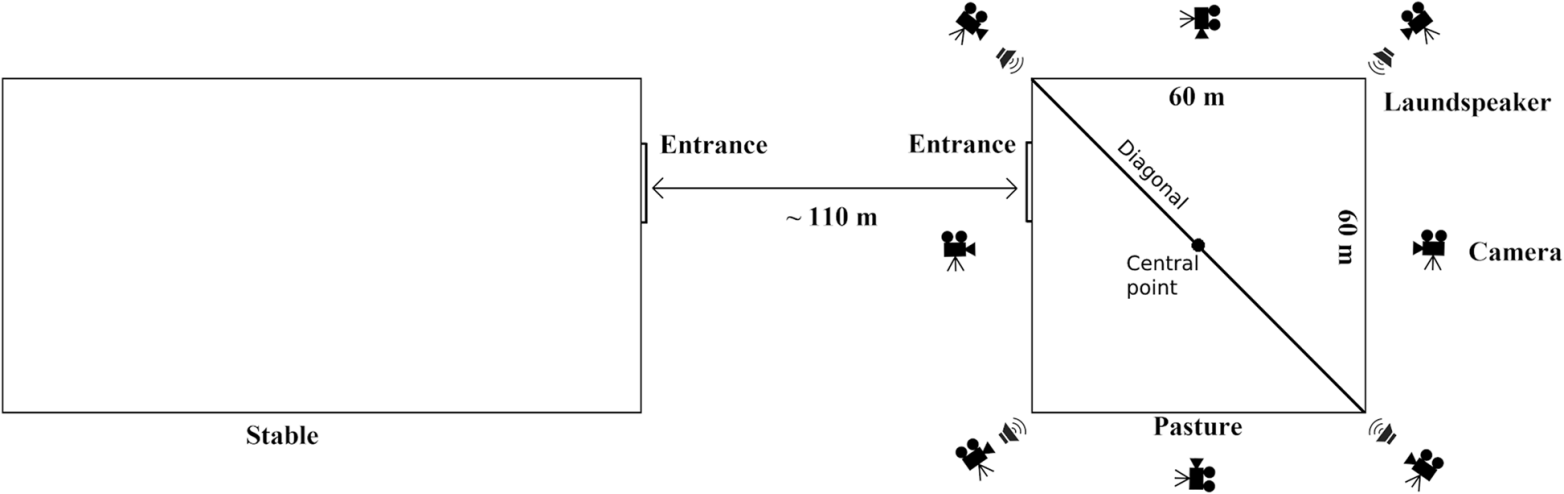
The location of the paddock, including the arrangement of loudspeakers and cameras

Each time, the sound signal was reproduced for five minutes. After the end of sound signal reproduction, the horses remained inside the fenced-off area. The sound was reproduced using two portable OneConcept CDC 100MP3 Hi-Fi devices. Each device was connected to two pairs of SONY SRS-XB12L Bluetooth loudspeakers located 1 m away from each corner of the fenced-off part of the pasture. The loudspeakers were not visible to the horses (Fig. 1). The levels and frequency ranges of the sounds were measured using a DT-8852 sonometer (CEM, Poland). The A-weighted LAeq was measured in decibels. The sounds produced by the thunderstorm and sea storm had similar values (61.4 dB for the storm and 58.4 dB for the thunderstorm), and a slightly higher level (68.5 dB) was recorded for the volcano. All three sounds had a maximum intensity at 1 kHz and were 55 dB, 60.7 dB and 69.4 dB for the thunderstorm, the sea storm and the volcano, respectively.

The course of the experiment was recorded using eight Panasonic HZ-900 digital cameras placed on tripods 110 cm above the ground, in the corners, and in the middle of each pasture walls.

On the days of the experiment, the air temperature was 23 ± 2.8 °C, atmospheric pressure: 995.64 ± 15.0 hPa, air speed: 0 ± 0,5 m/s.

In each part of the experiment, the analysis of the recordings was conducted in three 5-minute phases: 1) Before, means immediately before the sound signal reproduction, 2) During, means during the sound signal reproduction, and 3) After, means immediately after the sound signal reproduction. The analysis of the recordings was performed by one experienced person, who was blinded to which noise the studied horses had heard when assessing their behaviour.

### Analysed parameters

The distance between the horse and the central part / the paddock exit.

Based on the representative images extracted from camera footage: 30 s before the sound signal reproduction, representative of Before phase, 30 s before the end of sound signal reproduction, representative of During phase, and 30 s after the end of sound signal reproduction, representative of After phase, measurements of the distance between the horses and selected points of the pasture were taken. The position of each horse was marked on the images, and then the same position was identified on the paddock. In the next step, measurements were taken using a measuring tape with an accuracy of up to 0.1 m. During successive phases, in each part of the experiment, the following two distances were determined: 1) from the horse’s withers to the central point of the pasture, recognised as the most distant from the sound sources, i.e. located halfway along the diagonal line, i.e. at a distance of 30 m from the central point of each wall and 42.4 m from the corners; 2) the distance between the horse’s withers to the exit from the enclosure, i.e. the place located the nearest to the entrance to the stable.

### Behavioural observations

In each phase of the experiment, measurements were taken of the duration of the following elements of behaviour: walking, trotting and cantering, elements of behaviour categorised as “vigilance” (snoring, vocalisation, high head position, high tail position, sticking together), foraging (grazing), elements of behaviour categorised as “comfort” (playing, examining the surroundings, sniffing), elements of behaviour categorised as “maintenance of hygiene” (rubbing against objects, auto- or allogrooming, rolling) and resting. The frequency of expressing the following selected traits was also determined: snoring, vocalisation, high head position, high tail position, sticking together, rubbing against objects, auto- or allogrooming, rolling and resting. The characteristics of individual traits are presented in Table 7.

**Table 7:** Description of the behavioural traits under analysis

Category	Trait	Characteristics
Movement	Walk	Four-beat gait with large overlap times between the stance phases of the limb, and no period of suspension [40]
	Trot	Two-beat, symmetric, diagonal gait with phase suspension [40]
	Canter	Three-beat asymmetric gait with phase suspension. It is executed with either a right or left “lead” [40]
Vigilance	Snoring	Very short, raspy inhalation sound produced in a low alert context, such as investigating a novel object or obstacle [41]
	Vocalisation	Production of sound by means of a vocal apparatus of vertebrates [42]
	High head position	Neck raised over 45 degrees [43]
	High tail position	Fleshy part of tail outstretched horizontally or elevated above horizontal [43]
	Sticking together	Horses sticking together on the pasture
Foraging (grazing)		Occurs as a horse bites off and ingests grasses and forbs close to the ground [44]
Comfort	Playing	Type of self-enjoyment expression [44]
	Examining the surroundings	
	Sniffing	Standing with lowered head and nostrils within 10 cm of object [45]
Maintenance of hygiene	Rubbing against objects Scratching against an object	Includes using a hind foot to scratch another part of horse’s own body, or rubbing against various objects [46]
	Autogrooming or allogrooming	Usually head-to-shoulder or head-to-tail, grooming each other’s neck, mane, rump, or tail by gently nipping, nuzzling, or rubbing [42]
	Rolling	Dropping from standing to sternal recumbency, then rotating one or more times from sternal to dorsal recumbency, tucking the legs against the body [42]
Resting		It is characterised by a general lack of attention and a relaxed state and may occur in a standing position or in recumbency [44]

### Statistical methods

A statistical analysis was conducted using the SAS program (version 9.4, SAS Institute Inc. Cary, NC). Data distribution normality was assessed using the Kolmogorov-Smirnov test. For the data concerning the frequency of occurrence of the traits of snoring, vocalisation, high tail position, high head position, horses sticking together, rubbing against objects, auto- and allogrooming and rolling, the distribution was not normal (α = 0.05). In this case, a non-parametric Wilcoxon test was applied. The significance of differences between the phases in subsequent parts of the experiment was analysed by the Dwass, Steel and Critchlow-Fligner method. Data concerning the traits: distance (m) between the horses and the central point of the pasture/exit from the pasture, duration (s) of walking, trotting and cantering, vigilance, grazing and behaviour categorised as “comfort” and “resting” had a normal distribution (α = 0.05). In this case, the analyses were conducted using the GLM procedure while taking into account the effect of the phase factor and a subsequent part of the experiment and the interactions. The significance of differences between the mean values was determined by the Tukey test (HSD). The differences between the mean values were considered significant at *p* < 0.05. Due to the relatively small number of animals used in the study, the power analysis of the test was also performed. Assuring the test power at the level of 80%, the number of data was found to be enough to achieve the significance level as <0.05. The results were characterised using the mean value and standard deviation (SD).

## Supplementary Information

**Additional file 1.**

## Data Availability

The datasets used and analyzed during the current study are available from the corresponding author on reasonable request.
